# Depression and Anxiety Among Adolescents with Cancer: A Saudi Cross-Sectional Study

**DOI:** 10.3390/pediatric18050140

**Published:** 2026-10-16

**Authors:** Alyah A. Alqarni, Abdulhadi A. Alhabbad, Samah H. Alkhawashki, Hasan O. Batis, Areeb M. Altbenawi, Abdulrahman S. Alqumayzi, Badr M. Alqarni, Mais A. Alzarzor Alajami, Reham Y. Alturki, Ahmad H. Almadani, Alwaleed A. Alhazaa, Majed M. Alshehri

**Affiliations:** 1 Department of Psychiatry, Armed Forces Hospital Southern Region, Ministry of Defense Health Services, Khamis Mushait 62413, Saudi Arabia; dralia.psych@gmail.com (A.A.A.); a.qumayzi@gmail.com (A.S.A.); 2 Department of Psychiatry, King Saud University Medical City, King Saud University, Riyadh 11362, Saudi Arabia; aalhabbad@ksu.edu.sa (A.A.A.); badrm.alqarni@gmail.com (B.M.A.); 3 Department of Psychiatry, College of Medicine, King Saud University, Riyadh 11451, Saudi Arabia; salkhawashki@ksu.edu.sa (S.H.A.); ahalmadani@ksu.edu.sa (A.H.A.); 4 Oncology Center, King Saud University Medical City, Riyadh 11461, Saudi Arabia; hbatis@ksu.edu.sa; 5 Faculty of Medicine, Al-Arab University, Mukalla, Yemen; 6 Department of Psychiatry, Erada Mental Health Complex, Hail 55213, Saudi Arabia; a.altbenawi@gmail.com; 7 Department of Psychiatry, Johns Hopkins Aramco Healthcare, Dhahran 34465, Saudi Arabia; misoalajmi2000@gmail.com; 8 Department of Psychiatry, King Fahad Medical City, Riyadh Second Health Cluster, Riyadh 11525, Saudi Arabia; ryoalturki@gmail.com; 9 Adult Mental Health, King Abdulaziz Medical City, Ministry of National Guard Health Affairs, Riyadh 11426, Saudi Arabia; alwaleed1421@outlook.sa

**Keywords:** adolescent, anxiety, cancer, depression, Saudi Arabia

## Abstract

**Background/Objectives**: Adolescents undergoing treatment for cancer face both physical and psychological challenges, including depression and anxiety, yet local prevalence data remain limited in Saudi Arabia. The primary objective was to determine the prevalence of depressive and anxiety symptoms among adolescents with cancer at a tertiary center in Riyadh; a secondary, exploratory objective was to examine associations with socio-demographic and clinical factors. **Methods**: A cross-sectional study was conducted at a tertiary oncology center in Riyadh. Using convenience sampling, 86 adolescents aged 12–18 years with a confirmed tumor diagnosis completed a study-developed questionnaire and the validated Arabic PHQ-9 and GAD-7; analysis was restricted to the 59 participants with malignant disease. **Results**: Depressive symptoms of any severity were reported by 52.5% of participants (95% CI 40.0–64.7%), and anxiety symptoms by 37.3% (95% CI 26.1–50.0%). In secondary, exploratory analyses using a clinically meaningful threshold, both depression and anxiety were significantly more common among females than males (depression: 36.8% vs. 10.0%, *p* = 0.027; anxiety: 52.6% vs. 10.0%, *p* = 0.0006); anxiety was also significantly associated with age (highest at 14–16 years; *p* = 0.024). Most participants had no prior formal mental health diagnosis. **Conclusions**: Over half of adolescents with cancer in this Saudi sample experienced depressive symptoms, and over a third experienced anxiety symptoms; female gender was associated with both outcomes, and age with anxiety specifically, though these associations remain exploratory given the small sample. These findings support routine screening for all adolescents with cancer, with particular attention to female patients.

## 1. Introduction

Cancer is a significant health concern worldwide, affecting individuals across all age groups, including adolescents [1,2]. Approximately 400,000 children globally (aged 0–19 years) develop cancer each year [1,2,3,4], with rising incidence necessitating closer attention to its impact on this age group. In Saudi Arabia, childhood (aged 0–14) cancers accounted for 5.4% of all cancers, with leukemia being the most common (30.8%), followed by CNS tumors, Hodgkin’s lymphoma, and bone cancer [5]. Among adolescents and young adults (15–19 years), leukemia, lymphomas, and bone cancer predominate [6].

Depression is a common psychiatric disorder characterized by persistent sadness, loss of interest, and impaired functioning, and is a leading contributor to the global burden of disease across all age groups, including adolescents [7,8]. Global point prevalence of elevated depressive symptoms among adolescents rose from 24% (2001–2010) to 37% (2011–2020), with the Middle East, Africa, and Asia showing the highest rates, and with higher rates among females and older adolescents [9,10]. Depression during adolescence carries lasting implications for development and social functioning [11]. In Saudi Arabia, rates are comparable to global figures: 32.4% of 960 middle and secondary school students reported moderate to severe depression, with female gender, older age (16–19 years), and lower parental education as significant predictors [12,13].

Generalized Anxiety Disorder (GAD) involves excessive, persistent worry that is difficult to control, occurring most days for at least six months [7]. Pre-pandemic prevalence of clinically significant anxiety in a large Finnish adolescent cohort was approximately 11.6% [14], rising post-pandemic to an estimated 1 in 5 children and adolescents globally [15]. GAD is associated with disruption of amygdala-based emotion-regulation circuits [16] and can impair academic performance [17]. In Saudi Arabia, approximately one in two of 1723 adolescent males screened positive for an anxiety disorder [18].

Adolescents with cancer face a heightened risk of anxiety and mood disorders due to the physical and emotional demands of the disease and its treatment [19]. This risk is not uniform: most adolescents with cancer adapt well over time, while a meaningful subset—commonly estimated at roughly one quarter—experience clinically significant distress [20,21], reflecting the interplay of illness-related stressors with individual and social resistance factors such as age, sex, family support, and cultural context. Such factors are particularly relevant in Saudi Arabia, where strong extended-family support may act as a protective resource, while religious coping (e.g., Ruqyah, used by 20.3% of this sample) and cultural attitudes toward help-seeking may shape both the experience and reporting of distress. The primary aim of this study is to characterize the prevalence of anxiety and depression among adolescents with cancer at a tertiary oncology center; a secondary, exploratory aim is to examine factors associated with these outcomes, to inform targeted interventions relevant to the Saudi context.

## 2. Materials and Methods

Designed as a cross-sectional study, the research was carried out at the oncology outpatient departments and inpatient wards of a tertiary academic hospital in Riyadh, Saudi Arabia. Data collection took place between September 2024 and May 2025. Eligible participants were adolescents aged 12–18 years with a confirmed malignant-tumor (cancer) diagnosis who were either receiving active treatment or attending follow-up/survivorship care at the study site during this period, consistent with the population covered by IRB protocol E-24-9011.

The required sample size was estimated a priori using the single-proportion formula in Epi Info (CDC, Atlanta, GA, USA), based on prevalence estimates of 20% for depression and 14% for anxiety from a meta-analysis in pediatric and adolescent cancer patients [20] (Z = 1.96, e = 0.05), yielding targets of ~246 and ~185, respectively. As eligible adolescents at this single center were limited during the study period, we enrolled all those identified (86 completed questionnaires); of these, the final analytic sample reported here comprises the 59 participants with a malignant-tumor diagnosis (see Participants and Recruitment, above). Because the achieved sample was substantially smaller than planned, prevalence estimation was treated as the primary study objective, with point estimates reported alongside 95% confidence intervals; associations between symptom severity and demographic/clinical characteristics are reported as secondary, exploratory analyses, interpreted with attention to the resulting limits on precision (see Statistical analysis and Limitations). Consistent with current methodological guidance, a post hoc power analysis based on observed effect sizes was not performed, as such analyses do not provide meaningful additional information once effect estimates and their confidence intervals are known [22].

Participants were obtained by convenience sampling from the study site’s inpatient and outpatient oncology departments; the oncology case manager reviewed medical records to compile a list of eligible patients, irrespective of relapse or recurrence status. Eligible adolescents were aged 12–18 years with a confirmed malignant-tumor (cancer) diagnosis undergoing treatment or follow-up care, consistent with the population covered by IRB protocol E-24-9011.

**Exclusion criteria** were age outside this range, a benign (non-malignant) tumor diagnosis, or communication difficulties (cognitive impairment, difficulty reading Arabic, or health status impairing response) preventing questionnaire completion.

The oncology case manager’s records identified 130 adolescents for eligibility review. Of these, 24 were excluded before being approached (11 for age outside the 12–18 range; 13 for severe cognitive, communication, reading, or health difficulties precluding questionnaire completion), leaving 106 eligible adolescents who were approached.

Of those approached, 20 declined to participate or were unable to complete the questionnaire (e.g., fatigue, refusal, or acute medical instability during the clinical encounter), yielding 86 completed questionnaires (81.1% of those approached).

Of these 86 participants, 27 had a benign (non-malignant) tumor diagnosis, and hence were excluded from this analysis, yielding a final analytic sample of 59 adolescents with malignant-tumor (cancer) diagnoses.

**Figure 1:**
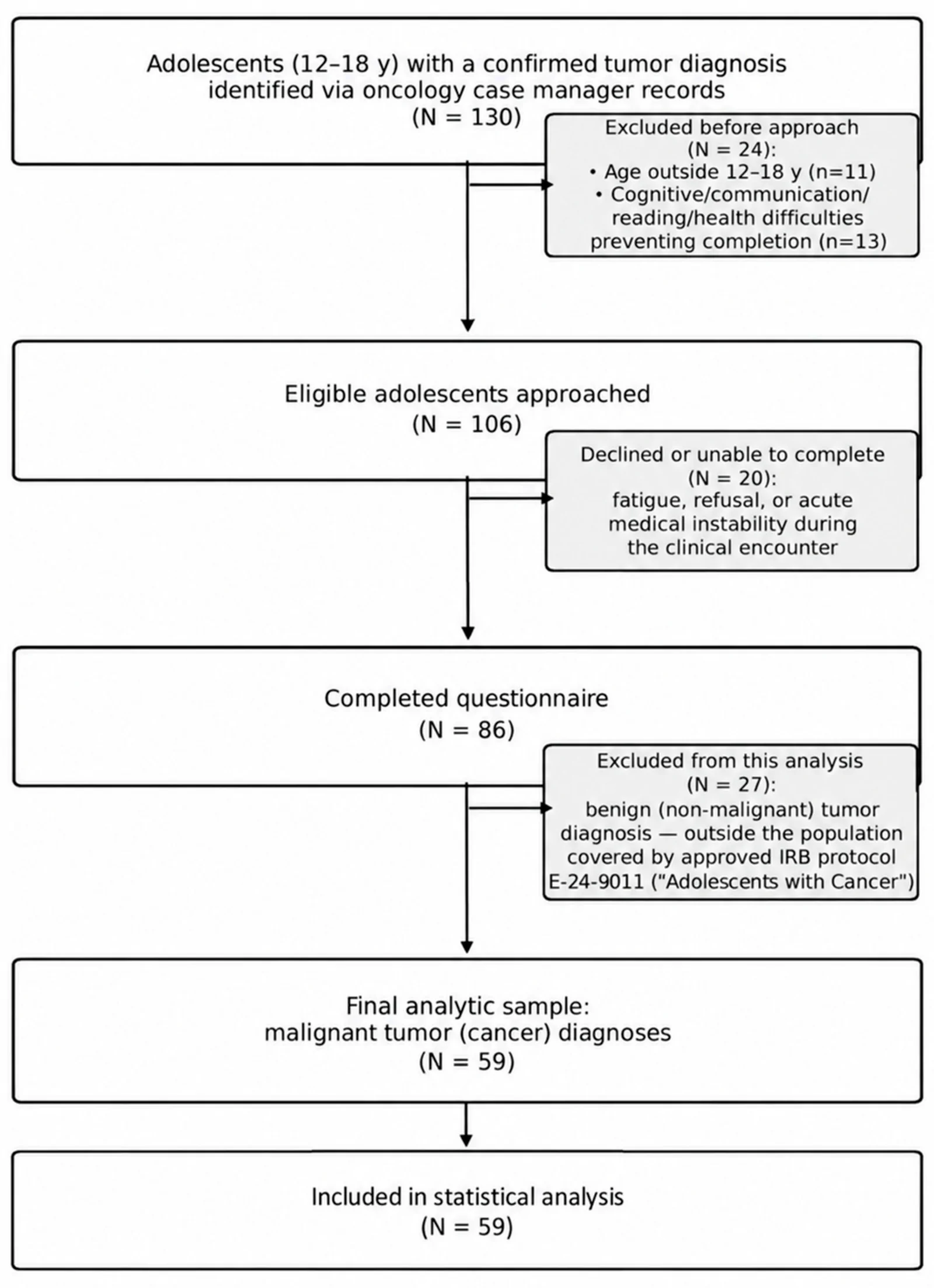
Participant flow diagram.

A STROBE-recommended participant flow diagram based on these figures is provided as Figure 1. Item-level missing data were minimal within the final analytic sample: no participant had a missing response on any PHQ-9 or GAD-7 item (59/59 complete on both instruments); no missing values were identified in the socio-demographic or clinical variables used in Table 1, Table 2, Table 3 and Table 4.

Eligible adolescents and guardians were approached in person during a clinic visit or admission and given a brief explanation before receiving a link/QR code to the electronic survey. Guardian consent and adolescent assent were obtained through a mandatory consent section at the start of the survey; participants could proceed only after confirming agreement. Participation was voluntary and did not affect medical care.

The structured electronic survey, completed in 10–15 min, collected no personally identifying data. Data collectors supported participants and guardians as needed. Data were anonymized and securely stored, accessible only to authorized team members.

The questionnaire covered age at diagnosis, tumor type, demographics, and treatment-related factors; items phrased about “the patient” were completed by adolescents themselves, consistent with the self-report PHQ-9/GAD-7. Tumor type (malignant or benign) was recorded for all participants who completed the questionnaire; participants with a benign tumor diagnosis were excluded from this analysis. Anxiety and depression were assessed using validated Arabic versions of the PHQ-9 and GAD-7, with strong psychometric properties in Saudi adolescents (Cronbach’s α = 0.857; sensitivity 89.5%, specificity 77.5% at cut-off of 11) [23].

The Arabic GAD-7 similarly shows strong reliability (α = 0.85) and validity in Saudi adult and adolescent populations, with a sensitivity of 86% and specificity of 83% at a cut-off of 10 [23,24].

In the present sample, internal consistency was excellent for both instruments (α = 0.87 PHQ-9; 0.90 GAD-7). Severity was categorized using standard PHQ-9 (minimal/mild/moderate/severe/very severe) and GAD-7 (none-minimal/mild/moderate/severe) bands. The original authors granted permission to use the validated Arabic translations, included as Supplementary Material Table S1.

Confidentiality and ethical standards were maintained throughout; participants and guardians were fully informed of the study’s aim, procedures, and voluntary nature. Ethical approval was granted by the Institutional Review Board of College of Medicine, at King Saud University (Protocol No. E-24-9011; Ref. No. 24/1602/IRB; Principal Investigator: Dr. Abdulhadi Ateeq Alhabbad; approved 15 September 2024, valid through 15 September 2025). The approved protocol covers adolescents with cancer. During data collection, 27 adolescents with benign (non-malignant) tumor diagnoses were also enrolled and completed the study questionnaire; as this falls outside the population covered by the approved protocol, these 27 participants were excluded from the present analysis, which is restricted to the 59 participants with a malignant-tumor (cancer) diagnosis, matching the approved protocol’s population exactly. No protocol amendment was required, as the analyzed population does not exceed the approved scope.

### Statistical Analysis

Data were analyzed using SPSS version 26.0. Descriptive statistics (mean, SD, frequencies, percentages) described quantitative and categorical variables. The primary analysis was descriptive: the prevalence of depressive and anxiety symptoms, overall and by severity band, is reported with Wilson score 95% confidence intervals. For secondary, exploratory analyses of associations between symptom severity and demographic/clinical factors, the five-level PHQ-9 and four-level GAD-7 severity bands used in the original analysis produced numerous sparse or empty cells (e.g., a single participant with no formal education), which the reviewers correctly noted made the resulting comparisons difficult to interpret reliably despite the technical applicability of exact tests. We therefore dichotomized each outcome at a clinically meaningful screening threshold consistent with the validated Arabic cut-offs reported above, using the moderate-or-above severity band as the threshold (PHQ-9 <10 vs. ≥10, closely approximating the validated Arabic PHQ-9 clinical cut-off of ≥11, which falls within this band; GAD-7 <10 vs. ≥10, matching the validated Arabic GAD-7 cut-off of ≥10 exactly), and re-examined associations using the Fisher–Freeman–Halton exact test on these collapsed tables. Full distributions across all original severity bands are retained in Table 2 and Table 3 for transparency, alongside the threshold-based comparisons. Because a large number of demographic and clinical factors were examined against two outcomes without adjustment for multiplicity, these analyses were treated as exploratory rather than confirmatory; no single pre-specified primary association was tested, *p*-values are not adjusted for multiple comparisons, and borderline results are interpreted cautiously rather than as confirmed effects. Non-significant results are not interpreted as evidence of no association, particularly for the smaller subgroups. A multivariable model was considered; given the small sample, sparse events, and the number of candidate covariates relative to the number of outcome events, a sufficiently stable adjusted model could not be supported by the available data, so associations are reported as unadjusted and exploratory, and interpreted accordingly. Significance was set at *p* < 0.05.

## 3. Results

The demographic characteristics of the participants are summarized in Table 1. The study included 59 adolescents with malignant tumors, aged between 12 and 18 years, with the largest proportion (57.6%) aged between 12 and 13 years, followed by 25.4% aged 17–18 years, and 16.9% aged 14–16 years. The majority were male (67.8%), and the largest group of participants had completed primary school education (35.6%), with only one participant reporting no formal education. Most participants reported that their parents were living together (88.1%), while 6.8% had divorced parents and 5.1% had lost one or both parents. Over half of the participants’ parents had a college degree (57.6%), and 83.1% of the participants resided in the central region of Saudi Arabia. A family history of mental disorders among first-degree relatives was reported by 3.4% of participants (n = 2), one reporting anxiety and one reporting obsessive–compulsive disorder.

Tumor-related factors within this malignant-tumor subgroup (N = 59) are summarized as follows. Nearly half of the participants (49.2%) were diagnosed with a tumor before age 12, and a similar proportion (49.2%) had been diagnosed for more than six months prior to the study. Most (78.0%) received initial treatment as inpatients, and 44.1% underwent their first treatment course within 1–6 months; 8.5% were treated for over three years. Comorbidities were present in 11.9% (most commonly H. pylori infection, n = 2, with asthma, seizures, diabetes, scoliosis, and favism each reported once). Most participants (67.8%) reported no anxiety or depressive symptoms following diagnosis, while 18.6% experienced symptoms within the first month, 10.2% between one and six months after diagnosis, and 3.4% more than six months after diagnosis (100% of participants accounted for).

**Figure 2:**
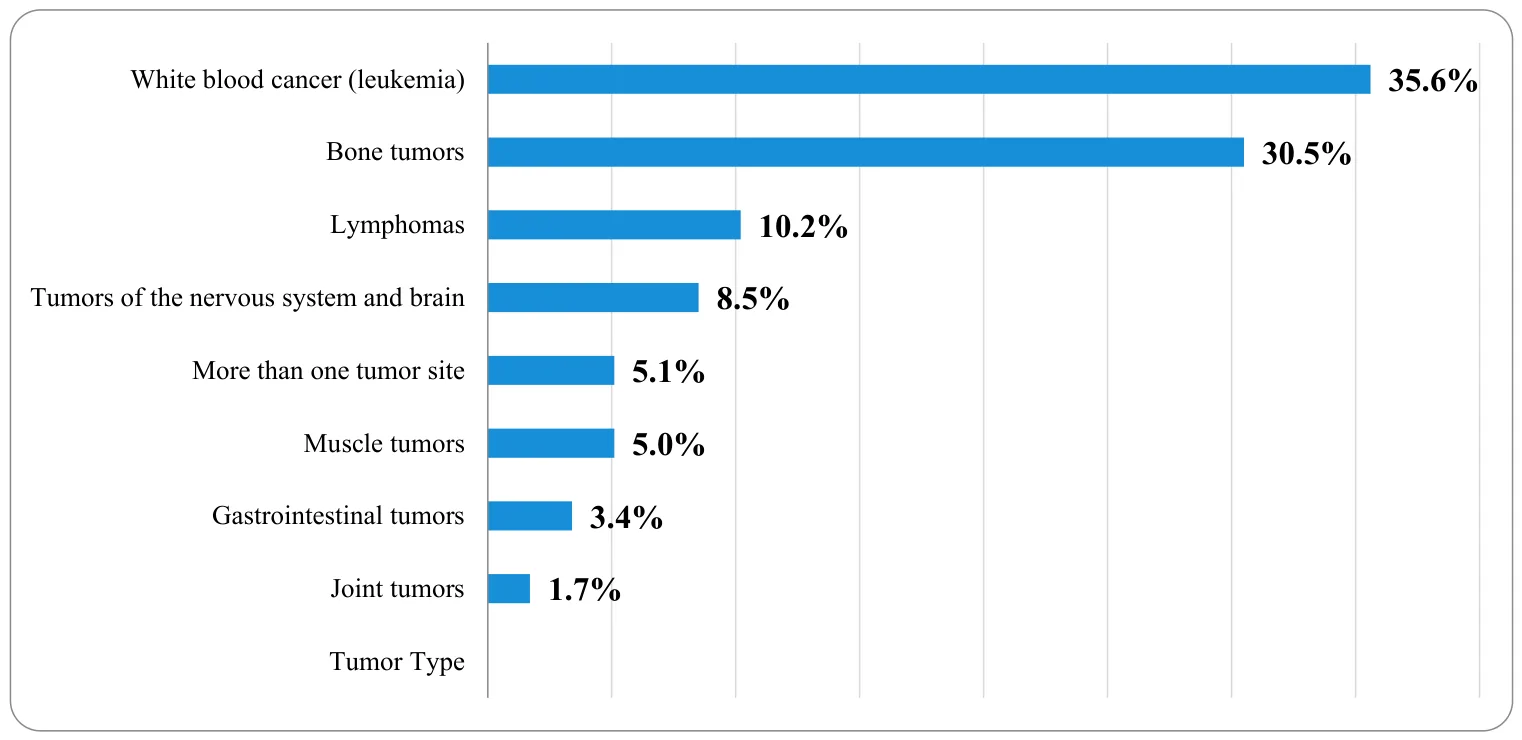
Distribution of tumor types among participants with malignant tumors (N = 59).

Figure 2 displays the distribution of tumor types among the 59 participants with malignant disease, categorized by anatomic/histologic site; categories are shown in descending order of frequency. Leukemia was the most prevalent (n = 21; 35.6%), followed by bone tumors (n = 18; 30.5%), lymphomas (n = 6; 10.2%), and tumors of the nervous system and brain (n = 5; 8.5%). Participants with tumors recorded at more than one anatomic site accounted for n = 3 (5.1%) and those with muscle tumors for n = 3 (5.0%); gastrointestinal tumors accounted for n = 2 (3.4%); and joint tumors accounted for n = 1 (1.7%). No papillomas were recorded among the malignant-tumor subgroup.

**Figure 3:**
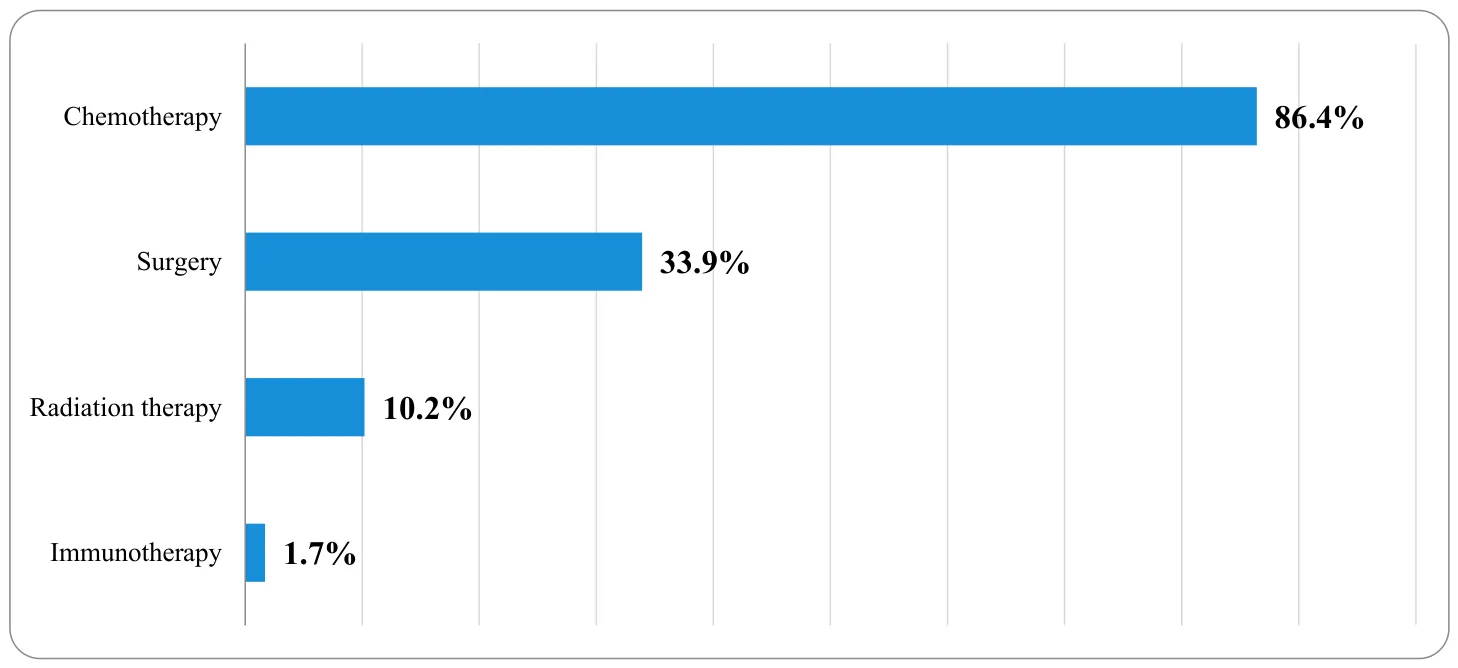
Treatment strategies.

As illustrated in Figure 3, chemotherapy was the most commonly used treatment modality, reported by 86.4% of the malignant-tumor subgroup (N = 59). Surgery was the second most used intervention (33.9%), followed by radiation therapy (10.2%) and immunotherapy (1.7%).

**Figure 4:**
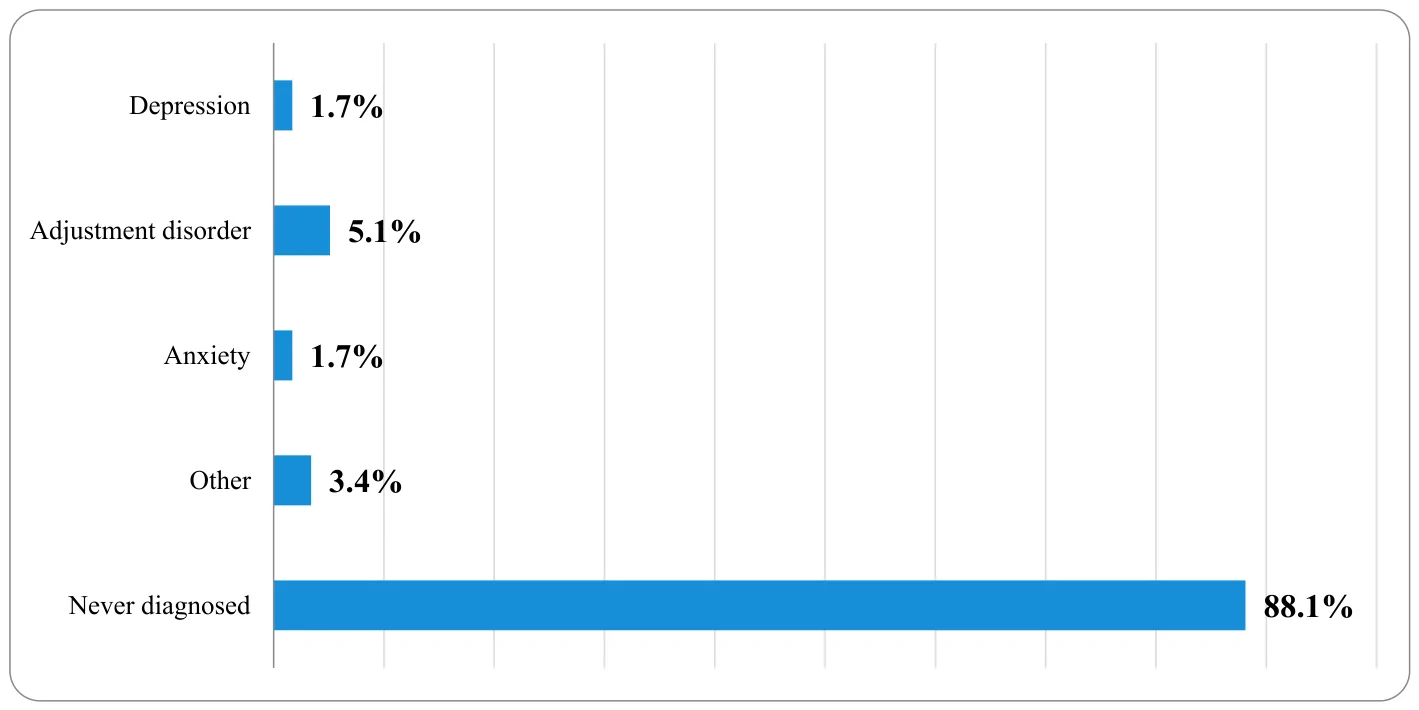
Prevalence of being diagnosed with mental disorders.

Figure 4 shows the reported prevalence of formal psychiatric diagnoses within the malignant-tumor subgroup (N = 59). The large majority (88.1%) had never been formally diagnosed with any mental disorder. Among the remaining 11.9%, adjustment disorder was the most common diagnosis (5.1%), followed by a remainder with another or more than one diagnosis (3.4%), and depressive disorder and anxiety disorders (1.7% each).

Data on therapeutic interventions and psychosocial aspects within this malignant-tumor subgroup (N = 59) are summarized as follows. Of the seven participants (11.9%) diagnosed with a mental disorder, four (6.8%) received pharmacological treatment and two (3.4%) were diagnosed but did not receive treatment; the remaining participants were not diagnosed with a disorder. Regarding alternative approaches, 20.3% used Ruqyah, and 3.4% used herbal remedies or supplements. Family relationships remained strong, with 57.6% describing them as excellent and 33.9% as good. Similarly, friendships were well maintained, with 47.5% rating them as excellent. Depression severity, measured by the PHQ-9, indicated that 47.5% had minimal depression, 33.9% had mild depression, and 18.6% reported moderate to very severe levels. For anxiety, measured by the GAD-7, 62.7% had no anxiety symptoms, 13.6% had mild anxiety, and 23.7% experienced moderate to severe levels.

Table 2 presents the full distribution of depression severity by demographic and clinical factors within the malignant-tumor subgroup (N = 59). In this exploratory, unadjusted analysis using the five-level PHQ-9 categorization, gender showed a nominally significant association with depression severity (*p* = 0.040), with females more likely to report moderate to very severe symptoms than males; age showed a non-significant trend (*p* = 0.067), and no significant differences were observed by educational level, parental marital status, parental education, or family psychiatric history. Given the small and unevenly distributed cell counts across five severity categories (e.g., a single participant with no formal education), these comparisons are difficult to interpret reliably; when depression severity was instead dichotomized at a clinically meaningful threshold (PHQ-9 ≥ 10; Table 4), the gender association remained significant (36.8% vs. 10.0%; *p* = 0.027), while age did not reach significance (*p* = 0.334). These associations should be regarded as exploratory hypotheses rather than confirmed risk factors, given the small subgroup sizes and lack of adjustment for multiple comparisons.

Lastly, Table 3 presents the full distribution of anxiety severity by demographic and clinical factors within the malignant-tumor subgroup (N = 59). In the exploratory, unadjusted five-level analysis, anxiety severity differed significantly by gender (*p* = 0.003), with females more likely to report moderate to severe anxiety than males; age showed a non-significant trend (*p* = 0.153), and no significant differences were observed by educational level, parents’ marital status, parents’ education, or family history of mental illness. When anxiety was dichotomized at a clinically meaningful threshold (GAD-7 ≥ 10; Table 4), the gender association remained significant and was, if anything, more pronounced (52.6% vs. 10.0%; *p* = 0.0006), and age also reached significance at this threshold (*p* = 0.024, highest in the 14–16 group), a pattern not apparent in the original five-level categorization; given the number of comparisons performed, this age finding should be treated as exploratory pending confirmation in a larger sample.

Because Table 2 and Table 3 contain several sparse or empty cells across the five-level PHQ-9 and four-level GAD-7 bands, we additionally re-examined each outcome using a clinically meaningful dichotomization at the validated screening threshold (PHQ-9 ≥ 10; GAD-7 ≥ 10), as recommended in peer review. Table 4 presents these threshold-based prevalence estimates with 95% confidence intervals for age and gender, the factors most examined in Table 2 and Table 3, within the malignant-tumor subgroup (N = 59); tumor type is no longer examined as a factor, as all analyzed participants have malignant disease (see Methods). Using the Fisher–Freeman–Halton exact test on these collapsed tables, the association between gender and clinically significant anxiety remained statistically significant and was stronger within this subgroup than in the originally submitted full sample (52.6% of females vs. 10.0% of males; *p* = 0.0006); gender was now also significantly associated with clinically significant depression (36.8% of females vs. 10.0% of males; *p* = 0.0272), an association that had not reached significance in the full sample. Age was significantly associated with clinically significant anxiety (11.8% at 12–13 years, 50.0% at 14–16 years, 33.3% at 17–18 years; *p* = 0.0235), while the age–depression association did not reach significance (*p* = 0.3344). Full exact *p*-values for the remaining factors in Table 2 and Table 3 are as follows: PHQ-9: educational level 0.583, parental marital status 0.843, parental education 0.448, family psychiatric history 1.000; GAD-7: educational level 0.625, parental marital status 0.400, parental education 0.255, family psychiatric history 1.000. None adjusted for multiplicity, and the family-history comparison is based on very few participants (n = 2 with a positive history) and should be interpreted with particular caution.

## 4. Discussion

Our findings—52.5% reporting depressive symptoms and 37.3% reporting anxiety, predominantly in the mild-to-moderate range—are broadly comparable to, if somewhat higher than, a prospective Omani study of 92 pediatric cancer patients, which reported initial rates of 43.5% and 56.5%, decreasing to 38.0% and 35.9% at 3–6 month follow-up [25]. This gap may reflect differences in assessment tools—the Omani study used SCARED, validated specifically for pediatric populations, versus our adult-validated GAD-7/PHQ-9—and in timing, since 49.2% of our sample was surveyed more than six months post-diagnosis, by which point acute distress often begins to resolve. Conversely, a global meta-analysis of ~2000 pediatric and adolescent cancer patients found lower pooled prevalence than ours (13.9% anxiety, 20.4% depression) [20], and a 16-country review reported 29% anxiety, 24% depression, and 32% distress among adolescent and young adult survivors [21]. Our comparatively higher rates may reflect differences in age range, tumor type, assessment tools, and population source—our sample was drawn entirely from active clinical care rather than survivorship cohorts. This regional gap underscores the value of routine, validated screening in Saudi adolescent oncology care.

In the exploratory, unadjusted analysis within this malignant-tumor subgroup, anxiety at or above the clinically meaningful threshold was significantly associated with age group (Table 4; *p* = 0.024), with the 14–16 group showing the highest proportion (50.0%), followed by the 17–18 group (33.3%) and the 12–13 group (11.8%); depression showed no significant association with age in this subgroup (*p* = 0.334). This age pattern for anxiety does not describe a simple monotonic trend—the middle age band shows the highest rate, not the oldest—and should be interpreted cautiously given the small subgroup sizes (n = 10 for the 14–16 group). Adolescents and young adults with cancer are generally recognized as being at elevated psychiatric risk relative to both healthy peers and older adult patients [26,27,28,29,30,31,32], though how this risk varies across early, middle, and late adolescence specifically is less established, and our cross-sectional, unadjusted data cannot distinguish a stable developmental pattern from a transient response tied to time since diagnosis or treatment phase. Larger, adequately powered, longitudinal studies are needed before age can be considered an established correlate of anxiety severity in this population.

Female gender was significantly associated with both clinically significant anxiety and clinically significant depression in this malignant-tumor subgroup (Table 4): 52.6% of females versus 10.0% of males met the anxiety threshold (*p* = 0.0006), and 36.8% of females versus 10.0% of males met the depression threshold (*p* = 0.027). The anxiety association was, if anything, stronger here than in the originally submitted full sample, making it the single most consistent finding of this study; the depression association did not reach significance in the full sample and emerged only once the analysis was restricted to the malignant-tumor subgroup. These findings are broadly consistent with the psycho-oncology literature showing that females with cancer across age groups tend to report higher anxiety and depression than males [33,34], and with population-based studies showing the same gender pattern among healthy adolescents [35]. This convergence—the same gender gap appearing both in the general adolescent population and specifically among adolescents with cancer—suggests that female gender may function as a fairly generalizable correlate of psychological distress rather than one specific to the oncology context, though as an unadjusted association from a single-center sample it should not yet be interpreted as an independent predictor, and the added burden of illness may still amplify an underlying predisposition. These findings may help clinicians anticipate and proactively screen for both anxiety and depressive symptoms in female patients with cancer, and future research—ideally with adjustment for co-occurring factors and examination of the biological, psychological, and sociocultural mechanisms underlying this gender disparity in adolescent oncology specifically—is recommended.

Formal psychiatric diagnoses were documented in only 11.9% of our sample, most commonly adjustment disorder, followed by depressive and anxiety disorders—a marked contrast with the substantially higher self-reported symptom rates on the PHQ-9 (52.5%) and GAD-7 (37.3%). This discrepancy likely reflects the nature of these tools as high-sensitivity screening instruments rather than diagnostic ones, though it is also consistent with broader evidence that depression and anxiety are under-recognized in patients with cancer generally [36]. Adolescents’ psychosocial needs can be underestimated at diagnosis, when clinical attention is understandably focused on the physical disease [37], and diagnostic overlap between tumor- or treatment-related symptoms (e.g., fatigue, sleep disturbance, appetite loss) and vegetative depressive symptoms can further obscure recognition [37]. Clinician hesitancy in prescribing SSRIs in pediatric populations, given FDA black-box warnings on suicidality, and limited access to mental health services are additional barriers [37]. Cultural norms around diagnosis disclosure in the region, where families sometimes prefer to shield adolescents from full diagnostic information, may also contribute to under-recognition. Notably, roughly half of participants diagnosed with a mental disorder received no formal pharmacological treatment, while religious and traditional coping resources (Ruqyah, herbal remedies) were drawn upon considerably more widely—suggesting these resources should be acknowledged, though not substituted for clinical care, in any psychosocial support pathway developed for this population. Overall, routine psychological screening and targeted support appear both feasible and necessary, and future work should validate Arabic-language tools that better distinguish tumor-related from depressive symptoms.

**Implications for practice**: These findings support three steps for adolescent oncology services in Saudi Arabia: (1) routine screening with validated tools (PHQ-9, GAD-7) for all adolescents with cancer at diagnosis and key treatment milestones, rather than restricting screening to presumed high-risk subgroups; (2) particular attention to female patients when prioritizing psychosocial referral, given the most consistent findings in this sample, while continuing to monitor age as a hypothesis meriting confirmation in larger studies; and (3) integration of culturally relevant coping resources, such as family support and religious coping practices, alongside conventional psychological care.

This study has strengths and limitations. Its focus on anxiety and depression in adolescents with cancer in Saudi Arabia addresses an area with limited existing research; using validated Arabic PHQ-9/GAD-7 instruments and examining socio-demographic and clinical associations provides insight beyond simple prevalence estimates.

Limitations include the small, single-center, convenience sample of adolescents with malignant tumors (n = 59), which constrains generalizability and reduces precision relative to the a priori target; several subgroup analyses—notably by family psychiatric history (n = 2 with a positive history) and educational level—involved small cell counts even after dichotomization, warranting particularly cautious interpretation.

Selection bias merits attention. Participants were recruited through convenience sampling during clinical encounters, which may over-represent adolescents well enough to attend appointments and complete an electronic, self-administered questionnaire, while under-representing those with more severe illness or logistical barriers to attendance. Adolescents with cognitive, communication, reading, or health-related difficulties that would have precluded questionnaire completion were excluded by design, possibly leading to underestimation of psychological distress in the source population.

The exploratory associations reported in Table 2, Table 3 and Table 4 were unadjusted for potential confounding among demographic and clinical variables, and a stable multivariable model could not be supported given the small sample and sparse events. Findings should therefore be regarded as hypothesis-generating rather than confirmatory and are not adjusted for multiple comparisons. Relapse/recurrence history and treatment intensity were not recorded, which may have influenced anxiety related to health status. Finally, the cross-sectional design precludes causal inference; longitudinal, multi-center studies with larger, randomly sampled populations and adjusted multivariable analysis are recommended to confirm these exploratory associations and track mental health over time.

## 5. Conclusions

This study examined anxiety and depression among adolescents aged 12–18 with cancer at a tertiary oncology center in Riyadh. We found that 52.5% (95% CI 40.0–64.7%) experienced depressive symptoms and 37.3% (95% CI 26.1–50.0%) reported anxiety symptoms, ranging from mild to severe, indicating that a substantial proportion of adolescents with cancer in this setting experience distress warranting attention. In secondary, exploratory analyses, female gender was associated with a higher likelihood of both clinically significant anxiety and clinically significant depression, the most consistent findings of this study; age was significantly associated with anxiety, though without a simple linear pattern across age groups. These associations are unadjusted, drawn from a reduced sample, and should be regarded as hypotheses for future confirmation rather than established correlates. These findings support routine, validated screening for all adolescents with cancer, with particular attention to female patients, rather than assuming risk is confined to specific demographic or clinical subgroups. Larger, longitudinal, multi-center studies with adjusted multivariable analysis are needed to confirm which factors are genuinely associated with psychological distress in this population.

## Figures and Tables

**Table 1:** Demographic factors of the participants with malignant tumors (N = 59).

Factor	Category	Count	Column N %
Age	12–13	34	57.6%
	14–16	10	16.9%
	17–18	15	25.4%
Gender	Male	40	67.8%
	Female	19	32.2%
Educational Level	No Education	1	1.7%
	Primary School	21	35.6%
	Intermediate School	20	33.9%
	High School	17	28.8%
Parents	Living Together	52	88.1%
	Divorced	4	6.8%
	One or Both Parents Are Dead	3	5.1%
Parents’ Educational Level	High School or Less	19	32.2%
	College	34	57.6%
	Post-Graduated Education	6	10.2%
Residency	Central Region	49	83.1%
	Northern Region	1	1.7%
	Southern Region	7	11.9%
	Western Region	2	3.4%
Family History of Any Mental Disorders in First-Degree Relatives	No	57	96.6%
	Yes	2	3.4%
Type of Mental Disorder (N = 2)	Anxiety	1	50.0%
	Obsessive–Compulsive Disorder	1	50.0%

**Table 2:** Association between depression severity (PHQ-9) and demographic factors, malignant-tumor subgroup (N = 59).

Factor	Category	Minimal n (%)	Mild n (%)	Moderate n (%)	Severe n (%)	Very Severe n (%)	*p*-Value
Age	12–13 (N = 34)	16 (47.1%)	14 (41.2%)	3 (8.8%)	1 (2.9%)	0 (0.0%)	0.067
	14–16 (N = 10)	6 (60.0%)	1 (10.0%)	1 (10.0%)	0 (0.0%)	2 (20.0%)	
	17–18 (N = 15)	6 (40.0%)	5 (33.3%)	3 (20.0%)	1 (6.7%)	0 (0.0%)	
Gender	Male (N = 40)	23 (57.5%)	13 (32.5%)	3 (7.5%)	1 (2.5%)	0 (0.0%)	0.040
	Female (N = 19)	5 (26.3%)	7 (36.8%)	4 (21.1%)	1 (5.3%)	2 (10.5%)	
Educational Level	No Education (N = 1)	1 (100.0%)	0 (0.0%)	0 (0.0%)	0 (0.0%)	0 (0.0%)	0.811
	Primary (N = 21)	8 (38.1%)	10 (47.6%)	2 (9.5%)	1 (4.8%)	0 (0.0%)	
	Intermediate (N = 20)	12 (60.0%)	5 (25.0%)	2 (10.0%)	0 (0.0%)	1 (5.0%)	
	High School (N = 17)	7 (41.2%)	5 (29.4%)	3 (17.6%)	1 (5.9%)	1 (5.9%)	
Parents	Living Together (N = 52)	26 (50.0%)	16 (30.8%)	6 (11.5%)	2 (3.8%)	2 (3.8%)	0.759
	Divorced (N = 4)	1 (25.0%)	2 (50.0%)	1 (25.0%)	0 (0.0%)	0 (0.0%)	
	One/Both Dead (N = 3)	1 (33.3%)	2 (66.7%)	0 (0.0%)	0 (0.0%)	0 (0.0%)	
Parents’ Education	High School or Less (N = 19)	9 (47.4%)	5 (26.3%)	4 (21.1%)	1 (5.3%)	0 (0.0%)	0.620
	College (N = 34)	14 (41.2%)	14 (41.2%)	3 (8.8%)	1 (2.9%)	2 (5.9%)	
	Post-Graduate (N = 6)	5 (83.3%)	1 (16.7%)	0 (0.0%)	0 (0.0%)	0 (0.0%)	
Family History	No (N = 57)	26 (45.6%)	20 (35.1%)	7 (12.3%)	2 (3.5%)	2 (3.5%)	0.672
	Yes (N = 2)	2 (100.0%)	0 (0.0%)	0 (0.0%)	0 (0.0%)	0 (0.0%)	

**Table 3:** Association between anxiety severity (GAD-7) and demographic factors, malignant-tumor subgroup (N = 59).

Factor	Category	No Anxiety n (%)	Mild n (%)	Moderate n (%)	Severe n (%)	*p*-Value
Age	12–13 (N = 34)	23 (67.6%)	7 (20.6%)	3 (8.8%)	1 (2.9%)	0.153
	14–16 (N = 10)	5 (50.0%)	0 (0.0%)	3 (30.0%)	2 (20.0%)	
	17–18 (N = 15)	9 (60.0%)	1 (6.7%)	3 (20.0%)	2 (13.3%)	
Gender	Male (N = 40)	30 (75.0%)	6 (15.0%)	3 (7.5%)	1 (2.5%)	0.003
	Female (N = 19)	7 (36.8%)	2 (10.5%)	6 (31.6%)	4 (21.1%)	
Educational Level	No education (N = 1)	1 (100.0%)	0 (0.0%)	0 (0.0%)	0 (0.0%)	0.847
	Primary (N = 21)	13 (61.9%)	4 (19.0%)	3 (14.3%)	1 (4.8%)	
	Intermediate (N = 20)	13 (65.0%)	3 (15.0%)	3 (15.0%)	1 (5.0%)	
	High school (N = 17)	10 (58.8%)	1 (5.9%)	3 (17.6%)	3 (17.6%)	
Parents	Living together (N = 52)	33 (63.5%)	7 (13.5%)	7 (13.5%)	5 (9.6%)	0.377
	Divorced (N = 4)	2 (50.0%)	0 (0.0%)	2 (50.0%)	0 (0.0%)	
	One/both dead (N = 3)	2 (66.7%)	1 (33.3%)	0 (0.0%)	0 (0.0%)	
Parents’ Education	High school or less (N = 19)	14 (73.7%)	2 (10.5%)	2 (10.5%)	1 (5.3%)	0.805
	College (N = 34)	18 (52.9%)	5 (14.7%)	7 (20.6%)	4 (11.8%)	
	Post-graduate (N = 6)	5 (83.3%)	1 (16.7%)	0 (0.0%)	0 (0.0%)	
Family History	No (N = 57)	35 (61.4%)	8 (14.0%)	9 (15.8%)	5 (8.8%)	1.000
	Yes (N = 2)	2 (100.0%)	0 (0.0%)	0 (0.0%)	0 (0.0%)	

**Table 4:** Prevalence of clinically significant depressive and anxiety symptoms (PHQ-9 ≥ 10/GAD-7 ≥ 10) by age and gender, malignant-tumor subgroup (N = 59), with 95% CIs (secondary, exploratory analysis).

Factor	Category	PHQ-9 ≥ Threshold n/N (%)	95% CI	GAD-7 ≥ Threshold n/N (%)	95% CI
Age	12–13 (N = 34)	4/34 (11.8%)	4.7–26.6	4/34 (11.8%)	4.7–26.6
	14–16 (N = 10)	3/10 (30.0%)	10.8–60.3	5/10 (50.0%)	23.7–76.3
	17–18 (N = 15)	4/15 (26.7%)	10.9–52.0	5/15 (33.3%)	15.2–58.3
Gender	Male (N = 40)	4/40 (10.0%)	4.0–23.1	4/40 (10.0%)	4.0–23.1
	Female (N = 19)	7/19 (36.8%)	19.1–59.0	10/19 (52.6%)	31.7–72.7

## Data Availability

The data supporting the findings of this study are available from the corresponding author upon reasonable request. The data are not publicly available due to privacy and ethical restrictions related to participant confidentiality.

## Supplementary Materials

The following supporting information can be downloaded at: https://www.mdpi.com/article/10.3390/pediatric18050140/s1, Table S1: STROBE Statement—Checklist of items to be included in reports of cross-sectional studies.

## Abbreviations

The following abbreviations are used in this manuscript:

CDC	Centers for Disease Control and Prevention
CNS	Central Nervous System
FDA	Food and Drug Administration
GAD	Generalized Anxiety Disorder
GAD-7	Generalized Anxiety Disorder-7
IRB	Institutional Review Board
PHQ-9	Patient Health Questionnaire-9
QR	Quick Response
SCARED	Screen for Child Anxiety-Related Emotional Disorders
SD	Standard Deviation
SPSS	Statistical Package for the Social Sciences
SSRIs	Selective Serotonin Reuptake Inhibitors
