# Supplementary material for: Depression and Anxiety Among Adolescents with Cancer: A Saudi Cross-Sectional Study
Source: Pediatr Rep. 2026 Oct 16;18(5):140. doi: 10.3390/pediatric18050140 (PMC13708576; doi:10.3390/pediatric18050140)
Supplement: Supplementary file 1 [file pediatrrep-18-00140-s001.zip › pediatrrep-4595860-supplementary.pdf]

Table S1. STROBE Statement — Checklist of items to be included in reports of cross-sectional studies

*Manuscript: "***Depression and Anxiety Among Adolescents with Tumors: A Saudi Cross-Sectional Study***" — updated to correspond to the revised manuscript submitted with this checklist.*

Section/Topic	Item #	Recommendation	Location in revised manuscript / status
Title and abstract	1	(a) Indicate the study's design with a commonly used term in the title or abstract.	Title ("Cross-Sectional Study"); Abstract, Patients and Methods
		(b) Provide, in the abstract, an informative and balanced summary of what was done and found.	Abstract (revised: prevalence stated as primary finding with 95% CIs; associations labeled secondary/exploratory)
Introduction — Background/rationale	2	Explain the scientific background and rationale for the investigation being reported.	Introduction, paragraphs 1-4
Introduction — Objectives	3	State specific objectives, including any prespecified hypotheses.	Introduction, final paragraph; Abstract Objectives (revised: prevalence = primary, associations = secondary/exploratory)
Methods — Study design	4	Present key elements of study design early in the paper.	Methods, paragraph 1
Methods — Setting	5	Describe the setting, locations, and relevant dates, including periods of recruitment, exposure, follow-up, and data collection.	Methods, paragraphs 1-2 (Sept 2024-May 2025)
Methods — Participants	6	(a) Give eligibility criteria, and the sources and methods of selection of participants.	Methods, paragraphs 2-3 (revised: recruitment paragraphgraph now reports the full funnel — 130 reviewed, 24 excluded before approach, 106 approached, 20 declined/unable, 86 analyzed — per reviewer comment 8)
Methods — Variables	7	Clearly define all outcomes, exposures, predictors, potential confounders, and effect modifiers. Give diagnostic criteria, if applicable.	Methods, paragraphs 5-8 (questionnaire, PHQ-9, GAD-7, cut-offs)
Methods — Data sources/measurement	8*	For each variable of interest, give sources of data and details of methods of assessment (measurement).	Methods, paragraphs 5-8

		Describe comparagraphbility of assessment methods if there is more than one group.	
Methods — Bias	9	Describe any efforts to address potential sources of bias.	Methods, paragraph 4 (assent/consent procedure); Limitations (revised: expanded discussion of selection bias mechanisms per reviewer comment 8)
Methods — Study size	10	Explain how the study size was arrived at.	Methods, paragraph 1 (a priori sample size calculation; achieved N=86 vs. targets of ~246/~185, with rationale for proceeding)
Methods — Quantitative variables	11	Explain how quantitative variables were handled in the analyses. If applicable, describe which groupings were chosen and why.	Statistical analysis (revised: explains both the original 5-/4-level severity bands and the new clinically meaningful dichotomization at validated cut-offs, per reviewer comment 4)
Methods — Statistical methods	12	(a) Describe all statistical methods, including those used to control for confounding.	Statistical analysis (revised)
		(b) Describe any methods used to examine subgroups and interactions.	Statistical analysis; Results, Table 2-3 text
		(c) Explain how missing data were addressed.	Methods, recruitment paragraphgraph (resolved from raw dataset: 0/86 missing PHQ-9/GAD-7 items; no missing socio-demographic values)
		(d) If applicable, describe analytical methods taking account of sampling strategy.	Methods, paragraph 1 (convenience sampling), Limitations
		(e) Describe any sensitivity analyses.	Statistical analysis / Table 4 (threshold-based reanalysis presented alongside the original categorization as a sensitivity analysis, per reviewer comment 4)
Results — Participants	13*	(a) Report the numbers of individuals at each stage of the study (e.g., eligible, examined, confirmed, included, completing follow-up, analyzed).	Methods, recruitment paragraphgraph and new Figure 1 (participant flow diagram): 130 reviewed → 106 approached → 86 analyzed
		(b) Give reasons for non-participation at each stage.	Methods, recruitment paragraphgraph: age outside range (n=11) and cognitive/communication/reading/health difficulties (n=13) excluded before approach; decline/fatigue/refusal/acute instability (n=20) after approach
		(c) Consider use of a flow diagram.	Added as new Figure 1; original Figures 1-3 (tumor type, treatment, psychiatric diagnosis)

			renumbered Figures 2-4 throughout the manuscript
Results — Descriptive data	14*	(a) Give characteristics of study participants and information on exposures and potential confounders.	Results, Table 1
		(b) Indicate number of participants with missing data for each variable of interest.	Methods, recruitment paragraphgraph: 0 missing on all variables analyzed (confirmed from raw survey dataset)
Results — Outcome data	15*	Report numbers of outcome events or summary measures.	Results, Tables 2-4
Results — Main results	16	(a) Give unadjusted estimates and, if applicable, confounder-adjusted estimates and their precision (95% CI). Make clear which confounders were adjusted for and why they were included.	Results (revised: 95% CIs added throughout); Statistical analysis (explains why an adjusted model was not fitted, per reviewer comment 7)
		(b) Report category boundaries when continuous variables were categorized.	Methods, Statistical analysis (PHQ-9/GAD-7 severity bands and clinically meaningful cut-offs defined)
		(c) If relevant, consider translating estimates of relative risk into absolute risk for a meaningful time period.	Table 4 (absolute prevalence with 95% CI reported by subgroup)
Results — Other analyses	17	Report other analyses done — e.g., analyses of subgroups and interactions, and sensitivity analyses.	Table 4; Results text (threshold-based reanalysis)
Discussion — Key results	18	Summarize key results with reference to study objectives.	Discussion, paragraph 1
Discussion — Limitations	19	Discuss limitations of the study, taking into account sources of potential bias or imprecision. Discuss both direction and magnitude of any potential bias.	Limitations (revised and expanded per reviewer comment 8)
Discussion — Interpretation	20	Give a cautious overall interpretation considering objectives, limitations, multiplicity of analyses, results from similar studies, and other relevant evidence.	Discussion (revised throughout to distinguish confirmed vs. exploratory findings, per reviewer comments 2, 4, 5, 6); Conclusions (revised)
Discussion — Generalizability	21	Discuss the generalizability (external validity) of the study results.	Discussion, Limitations (single-center, convenience sample)
Other information — Funding	22	Give the source of funding and the role of the funders for the present study.	Funding statement (back matter)
